# Influence of normal tide and the Great Tsunami as recorded through hourly-resolution micro-analysis of a mussel shell

**DOI:** 10.1038/s41598-021-99361-2

**Published:** 2021-10-06

**Authors:** Yuji Sano, Tomoyo Okumura, Naoko Murakami-Sugihara, Kentaro Tanaka, Takanori Kagoshima, Akizumi Ishida, Masako Hori, Glen T. Snyder, Naoto Takahata, Kotaro Shirai

**Affiliations:** 1 Center for Advanced Marine Core Research, Kochi University, Kochi, Japan; 2 Atmosphere and Ocean Research Institute, University of Tokyo, Chiba, Japan; 3 Graduate School of Science and Engineering, University of Toyama, Toyama, Japan; 4 Graduate School of Science, Tohoku University, Sendai, Japan; 5 Natural Sciences, Osaka Kyoiku University, Osaka, Japan

**Keywords:** Biogeochemistry, Natural hazards, Ocean sciences

## Abstract

We report here hourly variations of Mg/Ca, Sr/Ca, and Ba/Ca ratios in a Mediterranean mussel shell (*Mytilus galloprovincialis*) collected at the Otsuchi bay, on the Pacific coast of northeastern Japan. This bivalve was living in the intertidal zone, where such organisms are known to form a daily or bidaily growth line comprised of abundant organic matter. Mg/Ca ratios of the inner surface of the outer shell layer, corresponding to the most recent date, show cyclic changes at 25–90 μm intervals, while no interpretable variations are observed in Sr/Ca and Ba/Ca ratios. High Mg/Ca ratios were probably established by (1) cessation of the external supply of Ca and organic layer forming when the shell is closed at low tide, and (2) the strong binding of Mg to the organic layer, but not of Sr and Ba. Immediately following the great tsunami induced by the 2011 Tohoku earthquake, Mg/Ca enrichment occurred, up to 10 times that of normal low tide, while apparent Ba/Ca enrichment was observed for only a few days following the event, therefore serving a proxy of the past tsunami. Following the tsunami, periodic peaks and troughs in Mg/Ca continued, perhaps due to a biological memory effect as an endogenous clock.

## Introduction

Paleo-environmental parameters such as surface seawater temperature^1^, salinity^2^, pH^3^, and nutrient availability^4^ have been reconstructed by stable isotopes and trace element contents of marine calcium carbonate. The knowledge derived from coral skeletons, foraminifera tests, and bivalve shells is clearly important and useful in constraining global as well as local past climate changes^5^. There are a couple of analytical methods which permit trace element determination with both detection limits in the ppb to ppm range and a lateral spatial resolution better than 200 μm scale such as Inductively Coupled Plasma Atomic Emission Spectroscopy (ICPAES) with a micro-drill sampling technique, Inductively Coupled Plasma Mass Spectrometry (ICPMS) carried out with a laser-ablation sampling technique and Secondary Ion Mass Spectrometry (SIMS). If a lateral resolution of better than 3 μm is required, however, a laterally high-resolution ion microprobe, such as NanoSIMS, is a unique instrument applicable to the determination of minor and trace element abundances with reasonable precision and sensitivity^6^. Using a NanoSIMS, high-resolution analysis of a giant clam shell has provided an environmental proxy of the finest published temporal resolution Sr/Ca ratios produced by daily light cycles^7^ and Fe/Ca ratios produced by extreme weather events^8^; however, data at this short temporal scale of a bivalve shell appear to be occasionally influenced by unexpected biological controls on an organism’s physiology and not directly related to the ambient seawater temperature^9^. Further case-studies of high-resolution analysis are therefore required to confirm the applicability of this technique to marine carbonate geochemistry.

Normal tide is a physical process of sea level change derived from the combined gravitational forces of the Moon and the Sun, and the rotation of the Earth. The tidal elevation may be recorded in chemistry of mussel shells living in the intertidal zone, because these organisms show variable rates in shell growth, probably due to the time of air exposure during low tide^10,11^. There are only a few reports on geochemical proxies of past tidal cycles which use the shells of bivalves^12,13^ and analyses with an hourly-resolution have never been applied thus far. We present here Mg/Ca, Sr/Ca, and Ba/Ca ratios in a Mediterranean mussel shell with a few μm resolution, where Mg/Ca variation of a specimen may reflect daily or sub-daily tidal cycles. Furthermore, based on observation, we discuss an hourly Mg/Ca anomaly possibly derived from a great tsunami induced by the 2011 Tohoku earthquake.

## Results

### Age model and time resolution

**Figure 1:**
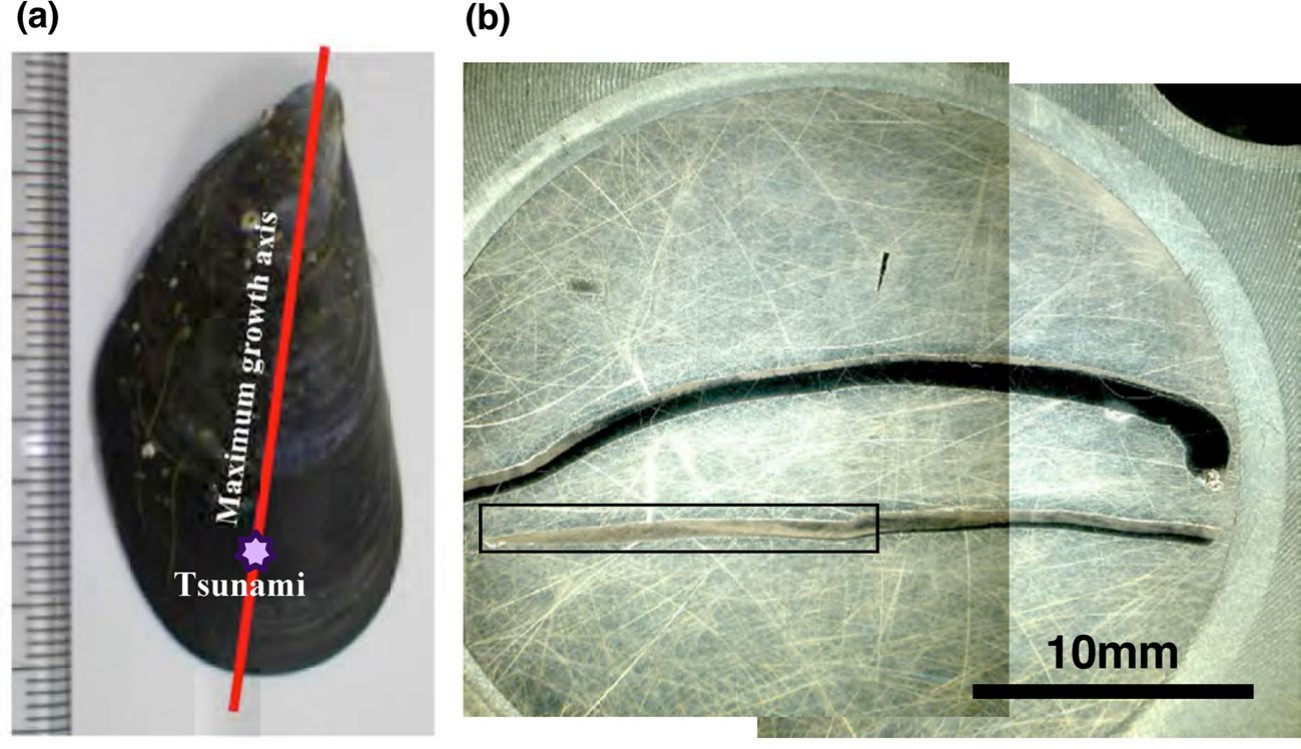
Photograph of the cut section and age-model of the mussel shell. (**a**) Photograph of *Mytilus galloprovincialis* collected at the Otsuchi bay, on the Pacific coast of northeastern Japan. A red line and purple star indicate the maximum growth axis and the approximate position of the great tsunami. (**b**) The cutting section mounted in a 2.54 cm diameter Araldite resin disk. Digital microscopy of the section stained by Mutvei’s solution for the squared area was shown in Figure S9.

**Figure 2:**
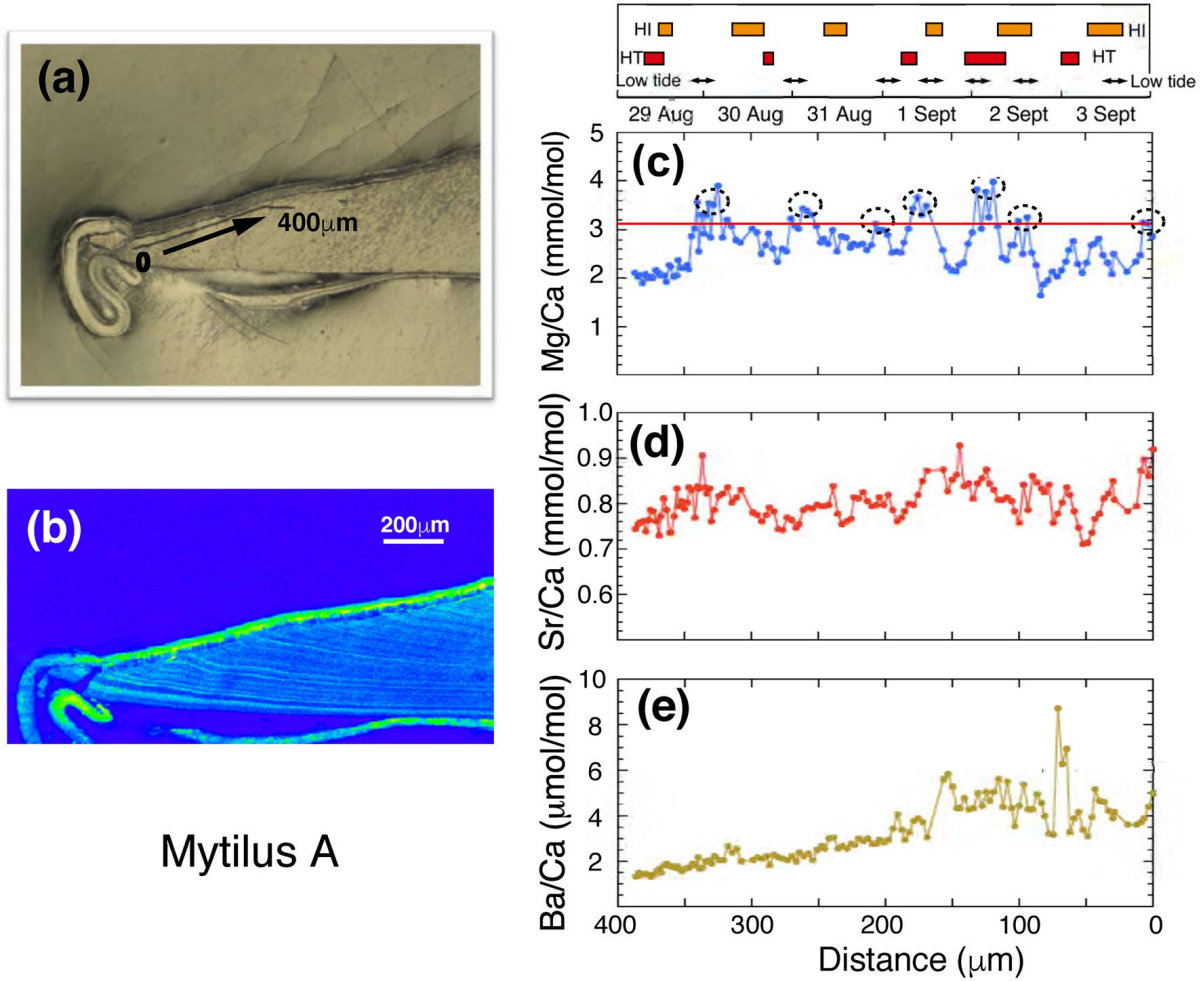
Analyzed position of the mussel shell sample and high-resolution profile of Mg/Ca, Sr/Ca and Ba/Ca ratios. (**a**) The trace of NanoSIMS analysis on the inner edge of the specimen. (**b**) A sulfur concentration map of the inner edge of the specimen measured by an EPMA. The approximate date is given in the top. (**c**) High-resolution profile of Mg/Ca ratios along the trace. A red line shows a threshold value. Ovals indicate values higher than the threshold. (**d**) High-resolution profile of Sr/Ca ratios along the trace. (**e**) High-resolution profile of Ba/Ca ratios along the trace. The simplified scales of the higher insolation (HI), higher temperature (HT), and lower tide period (double-edged arrow) are shown above the profiles for the references of environmental factors. The periods of HI, HT, and lower tide were certified based on the threshold values in Figure 5.

In the laboratory, soft tissue was removed from the shells and the center of one valve of the shell was cut from its umbo to the final ventral margin along the maximum growth axis (Fig. 1a). The shell was approximately 4.5 cm long, and a radial section was cut into two pieces along the center and both halves were mounted together in an Araldite resin disk of one-inch diameter (Fig. 1b) and polished (details are described later). An embedded section was stained by Mutvei’s solution, and the daily or bidaily growth line was counted to build the age model (see the Method section for details). The length of the collected specimen along the maximum growth axis is measured as 10.4 mm from the final ventral margin to the hypothetical position of the great tsunami on March 11th, 2011 by the reported age model^14^ (Fig. 1a). If we assume that the ventral margin was calcifying on the morning of September 6th, 2011 (sample collection date), the duration is 179 days and the average growth rate of this period becomes approximately 60 μm/day. Then the high-resolution mode, 2–3 μm interval, is equivalent to one hour in time resolution, which is probably the finest temporal resolution proxy reported to date in carbonate geochemical literature^7,12^. Figure 2a shows the trace of NanoSIMS analysis on the inner surface of the outer shell layer of the specimen. The starting point of analysis is located at roughly 120 μm inside from the final margin, so that date of the first measured point would be assigned to 3rd or 4th of September 2011. Figure 2b indicates a sulfur concentration map of the specimen measured by an EPMA. There is a significant number of condensed bands, possibly due to growth lines with enriched organic matter^16,17^. It is, however, difficult to count precisely.

At low-resolution mode of this study, 100 μm interval is equivalent with 1.7 days of time resolution in the part of the shell from the final ventral margin to the tsunami part, while the spot size of 20 μm may represent 8 h. It is practically impossible to measure all trajectories of approximately 45 mm from the ventral margin to the umbo by 2–3 μm intervals within a reasonable machine time. Therefore, a low-resolution analysis is necessary to cover the maximum growth axis of the whole shell.

### High resolution profiles of inner edge

Firstly, we report high resolution profiles of the inner edge. Figure 2a,b show the trace of NanoSIMS analysis and a sulfur content map. The positions of sulfur enrichment showed the positions of organic layers which were dye-stained area. A correlation diagram between the distance from the starting point and observed Mg/Ca ratios are shown in Fig. 2c. The Mg/Ca ratios vary substantially from 1.65 to 3.98 mmol/mol with the average of 2.67 ± 0.49 mmol/mol (1σ). There is an apparent cyclic change of the ratio, which will be discussed later together with a threshold value (a red line in Fig. 2c) and excess Mg data with dotted ovals. The Sr/Ca ratios are rather constant (Fig. 2d) and the variation of 0.802 ± 0.041 mmol/mol (1σ) is smaller than those of Mg/Ca ratio. The Ba/Ca ratios vary significantly from 1.34 to 8.74 μmol/mol with the average of 3.14 ± 1.37 μmol/mol (1σ) as indicated in Figure 2e. The variability of Ba/Ca ratios is much larger than that of Mg/Ca and Sr/Ca ratios and its pattern is monotonic decrease with a distance, including spikes and peaks which are not in phase with the cyclic change of the Mg/Ca ratios.

### High resolution profiles of the tsunami part

**Figure 3:**
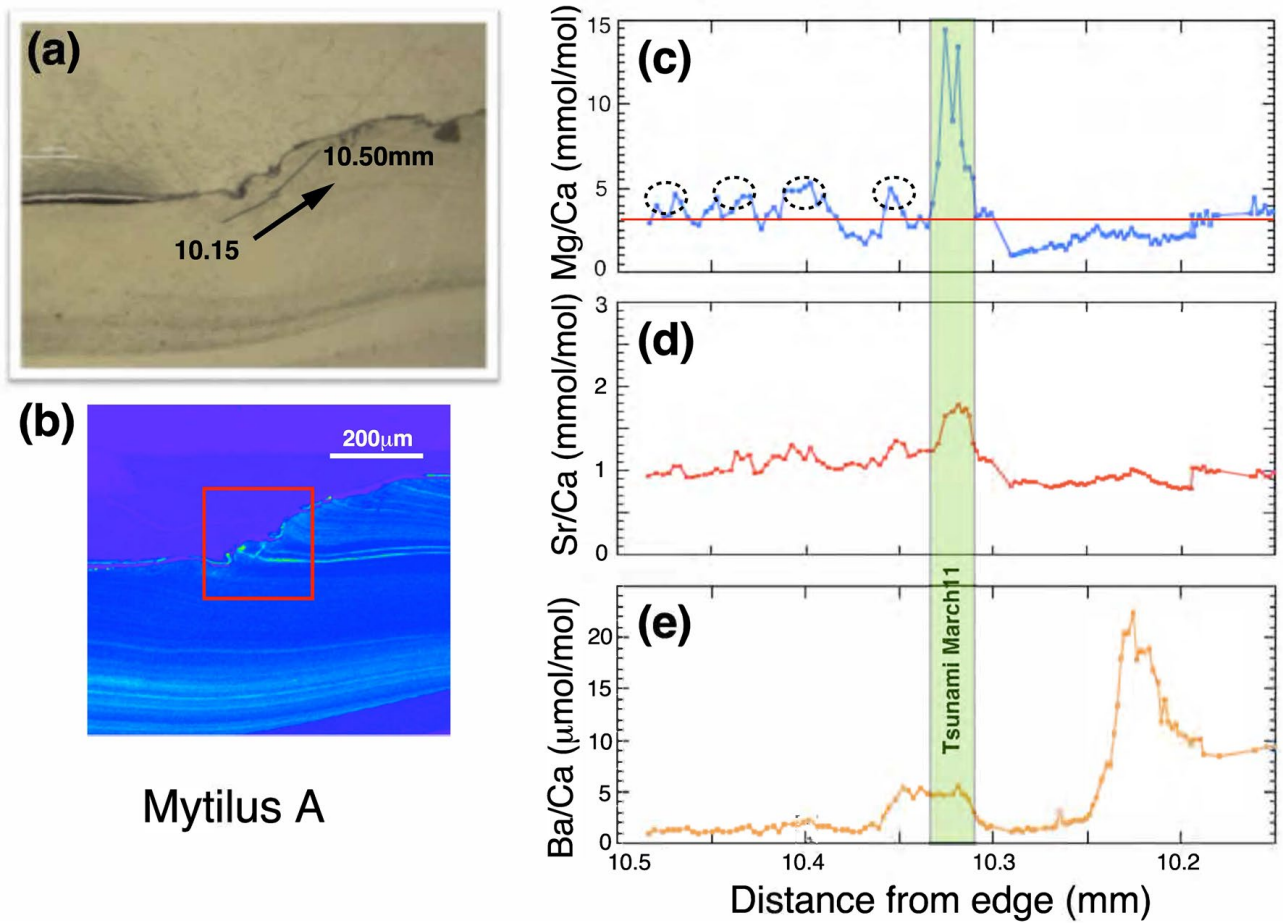
Analyzed position of the mussel shell sample and high-resolution profiles of Mg/Ca, Sr/Ca and Ba/Ca ratios. (**a**) The trace of NanoSIMS analysis on the tsunami part of the specimen. (**b**) A sulfur concentration map of the inner edge of the specimen measured by an EPMA. The approximate date is given at the top. (**c**) High-resolution profile of Mg/Ca ratios across the tsunami part. A red line shows a threshold value. (**d**) High-resolution profile of Sr/Ca ratios across the tsunami part. (**e**) High-resolution profile of Ba/Ca ratios across the tsunami part.

Secondly, we have selected the shell part of the specimen described above, corresponding to the period covering the great tsunami induced by the 2011 Tohoku earthquake based on the age model as stated above^14^. Figure 3a,b show the trajectory of NanoSIMS analysis in the part and a sulfur concentration map by EPMA, respectively. The Mg/Ca, Sr/Ca, and Ba/Ca ratios were measured by 2 μm spot analyses with 2 μm intervals over a distance of 10.15 mm to 10.30 mm starting at the inner edge. Those ratios between 10.33 mm and 10.48 mm were produced using 2 μm spot analyses with 3 μm intervals. Figure 3c indicates that the Mg/Ca ratios vary significantly from 1.03 to 14.4 mmol/mol with the average of 3.23 ± 1.92 mmol/mol (1σ). The threshold value (red line) in Figure 3c is at the same position of the inner edge diagram (Fig. 2c). Over the distance between 10.33 mm and 10.48 mm, there are the Mg/Ca peaks (dotted ovals), which are similar to observations of the inner surface of the outer shell layer. Significant enrichments of Mg are observed at distances between10.31 and 10.33 mm. The Sr/Ca ratios change from 0.78 to 1.79 mmol/mol with the average over this interval of 1.03 ± 0.21 mmol/mol (1σ, Fig. 3d). Variation between ratios is larger at the tsunami part than at the ventral margin, while the Sr/Ca data overall varies within 1σ deviation of the mean. A small peak in Sr/Ca is observed at a distance from 10.31 to 10.33 mm, coincident with the peak of Mg/Ca. The Ba/Ca ratios vary significantly from 0.90 to 22.4 μmol/mol with the average of 5.19 ± 5.46 μmol/mol (1σ) as shown in Figure 3e. There are two apparent Ba/Ca peaks; one is a small peak between 10.31 mm and 10.35 mm, largely coinciding but slightly earlier than that the Mg/Ca peak. The other is a large Ba/Ca peak from 10.19 to 10.24 mm, approximately 0.1 mm on the older side of the large Mg/Ca peak.

### Low resolution profiles of the whole shell

**Figure 4:**
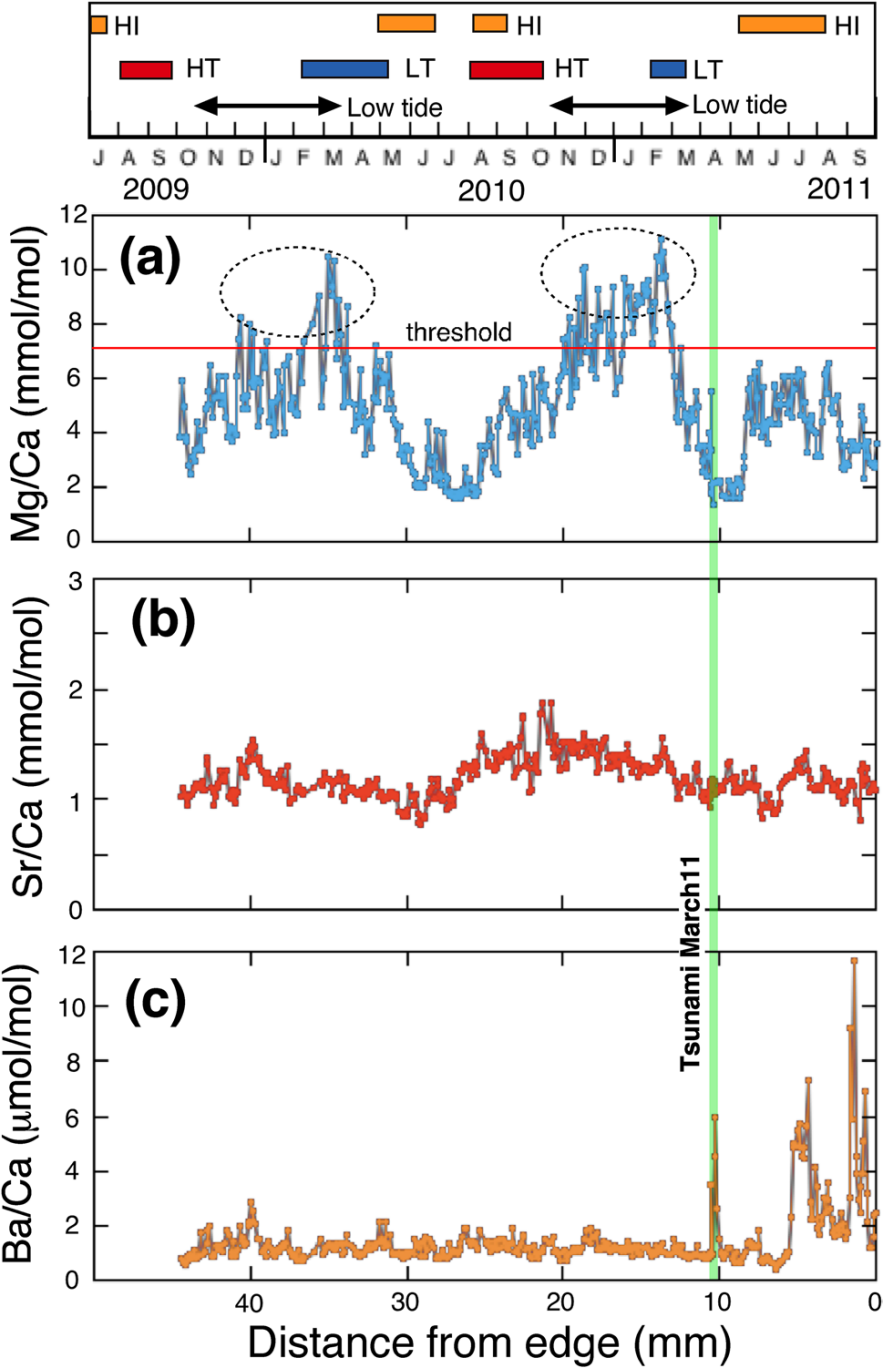
Low-resolution profiles of Mg/Ca, Sr/Ca and Ba/Ca ratios along the maximum growth line of the mussel shell sample. (**a**) Low-resolution profile of Mg/Ca ratios along the growth line. The horizontal red line and vertical green line show a threshold value and the date of the great tsunami, respectively. Ovals show the data higher than the threshold. (**b**) Low-resolution profile of Sr/Ca ratios along the growth line. (**c**) Low-resolution profile of Ba/Ca ratios along the growth line. The simplified scales of the higher insolation (HI), higher temperature (HT), and lower tide period (double-edged arrow) are shown above the profiles. The periods of HI, HT, and lower tide were certified based on the threshold values in Figure 7.

Finally, we show results from a low-resolution profile across the entire length of the shell (Fig. 4). The data shown covers the whole portion from the final ventral margin to the umbo of the shell along the maximum growth line. The Mg/Ca periodicity, coupled with previously published oxygen isotopic composition suggests that the life span of the specimen was from September 2009 to September 2011, approximately two years^14^. This specimen at the sampling time (September 6th, 2011) might be at a later stage in its life because the life span of *M. galloprovincialis* is 3–4 years^15^. A correlation diagram between the distance from ventral margin to the umbo and Mg/Ca ratio, Sr/Ca ratio, and Ba/Ca ratio are shown in Figure 4a–c, respectively. The Mg/Ca ratios vary significantly from 1.59 to 11.1 mmol/mol with the average of 5.35 ± 2.25 mmol/mol (1σ). The average is larger than those of inner shell surface, 2.67 mmol/mol and tsunami part, 3.23 mmol/mol measured by high-resolution mode. The threshold value (red line) of Figure 4a is calculated by the same method of inner shell surface (described later). The Sr/Ca ratios change from 0.77 to 1.89 mmol/mol with the average of 1.19 ± 0.18 mmol/mol (1σ). The average is larger than that of the inner surface, 0.802 ± 0.041 mmol/mol, while it is consistent with that of the tsunami part, 1.03 mmol/mol. There are two weak peaks with the distance of around 20.0 mm and 40.0 mm. The positions of peaks are not consistent with those of the Mg/Ca ratios and their amplitudes are much smaller. The Ba/Ca ratios vary significantly from 0.42 to 11.7 μmol/mol with the average of 1.50 ± 1.16 μmol/mol (1σ). The average is smaller than that of the inner edge, 3.14 μmol/mol (0–400 µm; Fig. 2e), and the tsunami part, 5.19 μmol/mol. The Ba/Ca ratios are constant and small until the tsunami, March 11, 2011, at the distance of 10.4 mm to 45.0 mm (Fig. 4c). A peak within the tsunami part (green band) is corresponding to a large peak in Figure 2e. There is a large variation of Ba/Ca ratio between 0 and 5 mm (Fig. 4c).

## Discussion

### Factors controlling high-resolution data in mussel shells

**Figure 5:**
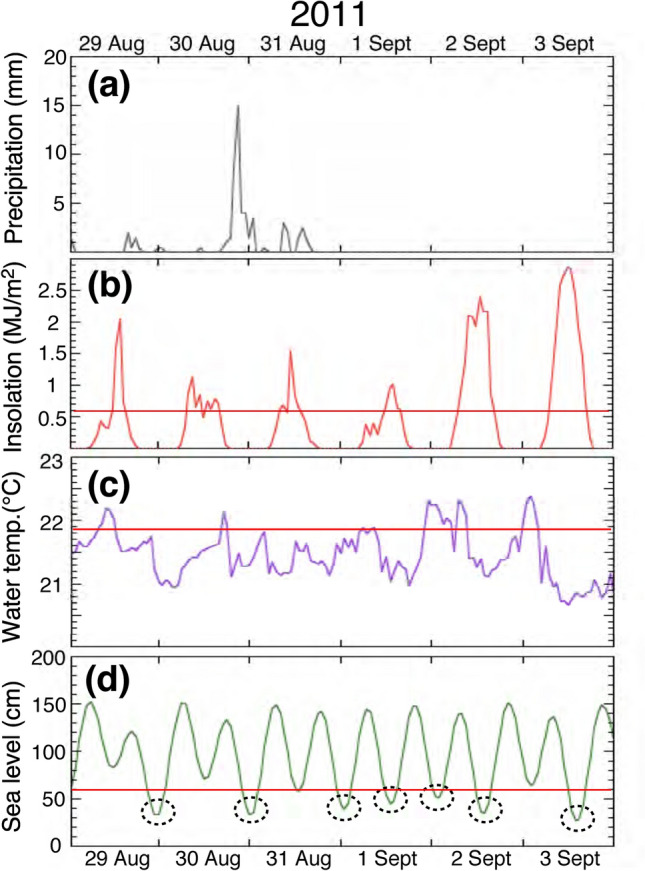
Environmental data from August 29th to September 3rd, 2011 in the studied region. (**a**) A record of hourly precipitation in Miyako Bay, located 40 km north of the sampling site. (**b**) A record of hourly insolation in Morioka city, 75 km northwest of the sampling site. A red line shows an arbitrary threshold value (0.6 MJ/m^2^). (**c**) A record of hourly surface seawater temperature in Hirota Bay, 45 km southwest of the sampling site. The data from an open database provided by a local government (https://www.suigi.pref.iwate.jp/teichi). A red line shows a threshold value that is the temperature one sigma higher than the average. (**d**) A record of hourly sea level change calculated by astronomical data in Kamaishi Bay, 9 km south of the sampling site. The all data from the nearest observation point to the sampling site, which are provided from the Japan Meteorological Agency (https://www.jma.go.jp/jma/menu/menureport.html). A red line shows an arbitrary threshold value (60 cm).

We compare high resolution data from the inner surface of the outer shell layer because they are close to the sampling date and, as such, the age model is the most reliable. Following the great tsunami on March 11th, 2011, meteorological and oceanographic data are not available in Otsuchi Bay, because the monitoring stations were seriously damaged. Figure 5a–d show a record of the hourly precipitation in Miyako Bay, located 40 km north of the sampling site (Fig. S1), as well as the variation of hourly insolation in Morioka city, 75 km northwest, the surface seawater temperature in Hirota Bay, 45 km southwest, and sea level change calculated by astronomical data in Kamaishi Bay, 9 km south, respectively, from August 29th to September 3rd, 2011. These collectively comprise the environmental data set, obtained in closest proximity to Otsuchi Bay.

Previous sclerochronological studies (the research of physical and chemical variations in the accretionary hard tissues of invertebrates) have revealed that mussel shells may be characterized by tidally-controlled growth patterns, perhaps due to the sea level changes^10,11^. Gene expression profiling of mussels has also revealed that tidal and circadian rhythms occur in a simulated intertidal environment^18^. In order to compare Mg/Ca ratio with sea level, we tentatively draw a threshold line (red color) at 3.16 mmol/mol of Mg/Ca ratio in Fig. 3c and that at 60 cm sea level (red color) in Figure 5d. There is no biochemical and physiological basis of the value of 3.16 mmol/mol, but simply one sigma higher than the average. On the other hand, the height of 60 cm may be hypothetically related with the position of the specimen relative to the sea level when the mussel was attached to a quay wall, even though the mussel was located always underwater after the 100 cm of land subsidence caused by the 2011 Tohoku earthquake.

**Figure 6:**
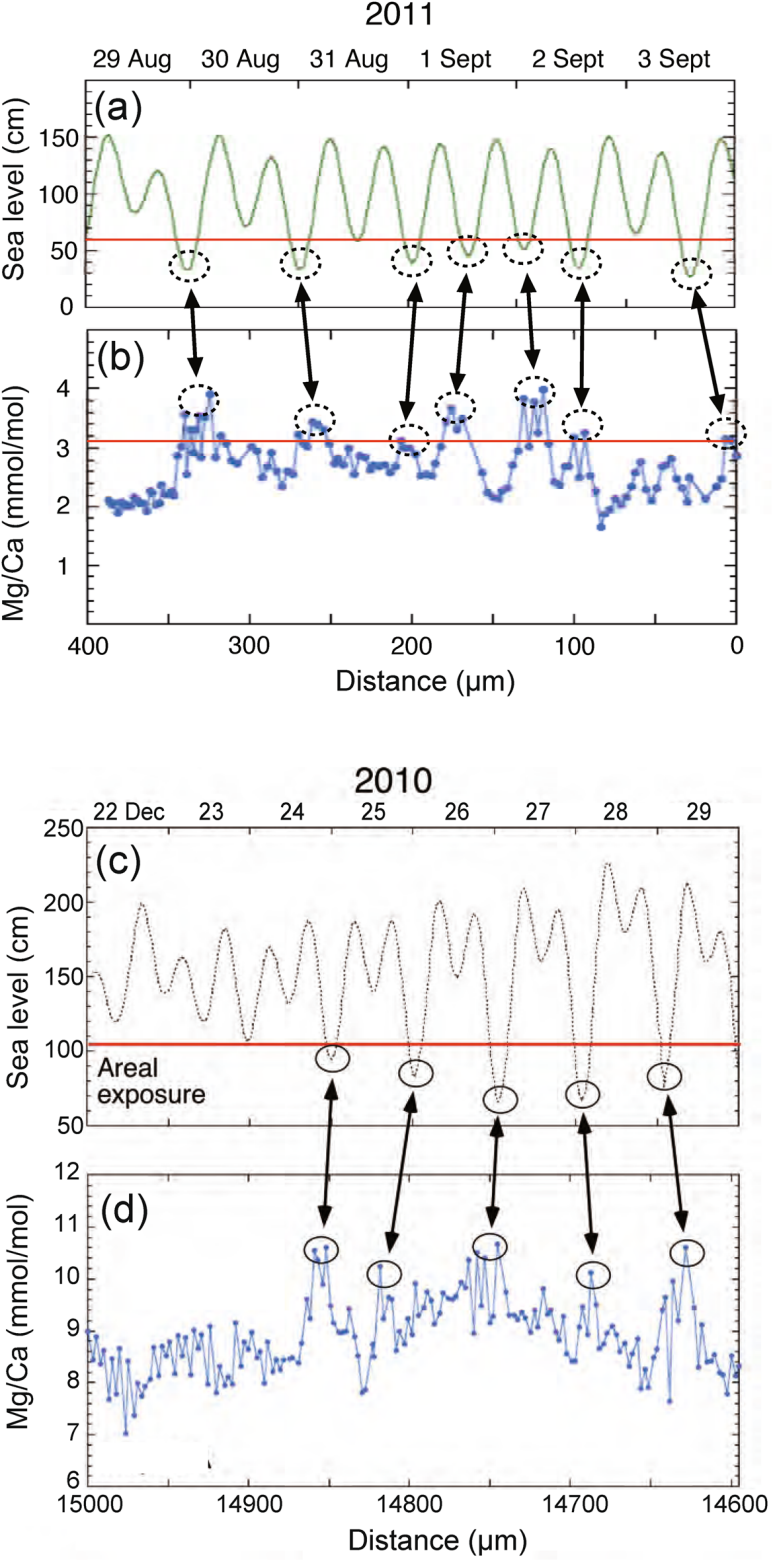
Relationships between sea level change and high resolution Mg/Ca ratio of the specimen A. The profile (**a**) comes from Figure 5c, and the profile (**b**) comes from Figure 2c. The profiles (**c**,**d**) are from the other measurement site (14.6–15.0 mm) of the specimen A. In both sites, correspondences between Mg/Ca peaks and sea level gouge were observed.

The seven Mg/Ca peaks higher than the threshold (dotted ovals in Fig. 2c) are apparently corresponding to seven sea level gauge readings lower than the threshold (dotted ovals in Fig. 5d), which summarized in Figure 6a,b. The similar correspondence between Mg/Ca peaks and sea level gouge was observed at other measurement sites (Fig. 6c,d). We estimate the distance from the center of each oval for seven Mg/Ca peaks and also calculate the elapsed time at each gorge of sea level by total hours before 0 am of September 4th. Then the distance is plotted against the elapsed hours in Figure S2a. The errors assigned to the distance are approximately 10 μm estimated from the average value of width and position of the seven peaks over the threshold (Fig. 2c). The errors (~ 10 µm) may not be precise but reasonable in the allowable range. There is a significant linear relationship between these parameters, suggesting that the proposed model of tidal control of Mg/Ca ratio is valid. A least square method gives the best fit of *y* = − 7 ± 19 + (2.84 ± 0.28) *x* where the error is 2σ, R^2^ = 0.988, and MSWD = 1.59. The averaged hourly increment of mussel shell would be 2.84 μm, similar to the analytical interval of 2–3 μm in high resolution mode, which is consistent with the resolution of one hour estimated above section. Note that the specimen was not collected from the intertidal zone at the time. The temporal variation of Mg/Ca ratio is not the direct consequence of air exposure. The variation may be due to either biological memory of specimen as endogenous clock or variation in intensity and direction of surface currents related to tides^19^.

We should discuss the relation between Mg/Ca ratios and other environmental parameters. There is no simple correlation between the Mg/Ca ratio and insolation (Fig. 5b). When we take an average seawater temperature of 21.45 ± 0.40 °C, there are five peaks based on the temperature one sigma higher than the average (a red line in Fig. 5c). These five peaks are not corresponding to the observed Mg/Ca ratio, suggesting that there is no apparent temperature control. Accordingly, the major control of Mg/Ca ratio of the mussel shell is probably attributable to the ambient tidal cycle.

This hypothesis is robust and significant because the cyclic change of Mg/Ca ratios is reproduced in the second set of high-resolution analysis of a different Mytilus individual (specimen B) living at the same location during the same time period (Fig. S3a) where the threshold of Mg/Ca ratio is again one sigma (0.55 mmol/mol) higher than the average (3.93 mmol/mol). The time and space relationship of Mg/Ca peaks and the tidal cycle is investigated and given in Figure S2b, where the best fit line is *y* =  + 2 ± 15 + (2.56 ± 0.22) *x* where the error is 2σ, R^2^ = 0.987 and MSWD = 1.41. The average growth rate of this specimen would be 2.56 ± 0.22 μm/h, consistent with that of specimen A, 2.84 ± 0.28 within the error.

There is no interpretable correlation between the Sr/Ca ratio (Fig. 2d) and sea level (Fig. 5d), which is verified and confirmed by the second set of data (Fig. S3b). This suggests that there is no tidal control on the Sr/Ca ratio. We cannot find any relationship between the Sr/Ca ratio and seawater temperature as well as precipitation (Fig. 5a) and insolation (Fig. 5b). This characteristic is consistent with the second data set (Fig. S3b).

The temporal variation of Ba/Ca ratio is complicated (Figs. 2e and 3e). The monotonic decrease of the ratio from distance 150 μm to 380 μm is different from temporal variations of Mg/Ca and Sr/Ca ratios. We find a large spike-type peak of Ba/Ca ratio at 70 μm and a small one at 150 μm. The second data set shows a similar decrease from 200 μm to 360 μm with a spike peak at 130 μm (Fig. S3c). There is no component with gradual decrease or increase in insolation, water temperature and sea level during the time period corresponding to the Ba/Ca variation. The increase of Ba/Ca ratio with time in the mussel shell may reflect the background variation of seawater Ba/Ca ratio^20^ or the changing physiology of the organism through its life^21^.

### Factors controlling low-resolution data in mussel shells

**Figure 7:**
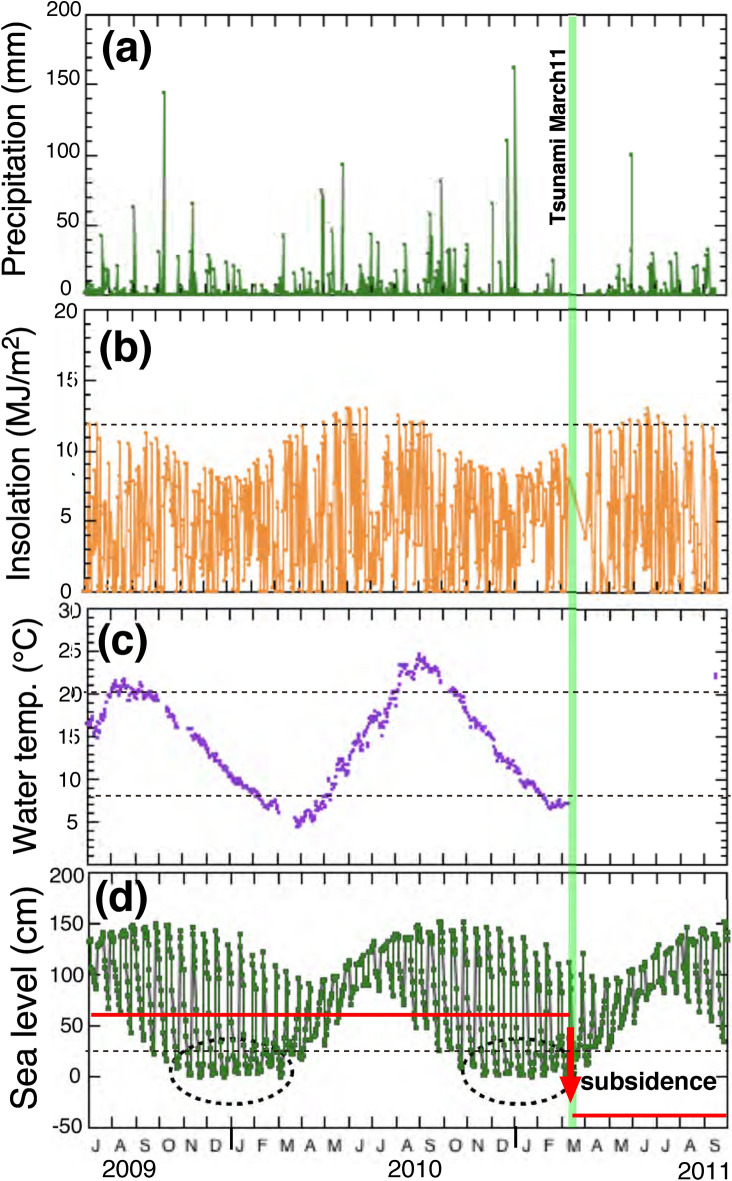
Environmental data from June 2009 to September 2011 in the studied region. (**a**) A record of daily precipitation in Miyako Bay, located 40 km north of the sampling site. (**b**) A record of daily insolation in Morioka city, 75 km northwest of the sampling site. A dashed line shows an arbitrary threshold value (12 MJ/m^2^). (**c**) A record of daily surface seawater temperature measured in the sampling site of Otsuchi Bay. Dashed lines show arbitrary threshold values (20 °C and 8 °C). (**d**) A record of daily sea level change calculated by astronomical data in Kamaishi Bay, 9 km south of the sampling site. Ovals show the low tide during the period. The profiled was descrived by using a soft ware “TIDE for WIN” (http://fmie.cside7.com/program/tide.html). A dashed line shows an arbitrary threshold value (25 cm).

It is important to verify the knowledge derived from high-resolution analysis by low-resolution data, because environmental control on minor and trace elements in high-resolution mode was somewhat different from that of low-resolution in the case of foraminifera^22^. On the other hand, there are insolation controls on Sr/Ca ratios of giant clam shells in both high-resolution and low-resolution observations^7^. Figure 7a–d show a record of daily precipitation in Miyako Bay (Fig. S1), variation of daily insolation in Morioka city, surface seawater temperature in Otsuchi Bay referred from previous work^14^, and sea level change calculated in Kamaishi Bay, respectively, from September 2009 to September 2011. The red line in Figure 7d shows the hypothetical height of the specimen relative to sea level. It was 100 cm lowered by the coseismic subsidence induced by the 2011 Tohoku earthquake as indicated by an arrow. Then the mussel was located always underwater after the earthquake.

In accordance with the previous pioneering work of Dodd on the Mg/Ca ratios of mussel shells as a temperature proxy^23^, we conducted a couple of measurements on mussel shells with low-resolution mode (30 μm–300 μm). Seawater temperature control on the Mg/Ca ratio has been observed in some previous case studies^24,25^, but was not well reproduced in another case^17^. The discrepancies are partly due to the unknown biochemical and physiological mechanism of Mg incorporation into mussel shells. In the present study, there are two broad peaks of Mg/Ca ratios higher than the threshold in the temporal variation, located from 13 to 20 mm and from 33 to 41 mm (Fig. 4a). The latest peak (from 0 to 8 mm) is lower than the threshold. On the other hand, the lowest sea levels were observed in winter from November to February in each year (see dotted ovals in Fig. 7d). These winter low tides may be corresponding to two broad Mg/Ca peaks in the temporal variation, suggesting that the major control of Mg/Ca ratio may be due to the ambient tidal cycle. Irregular variation of Mg/Ca ratios after the earthquake may be due to the effect of regional coseismic land subsidence on the sample site, since the mussel was always located underwater after the earthquake. There are significant phase shifts between peaks of Mg/Ca ratio and insolation (Fig. 7b), and those of the ratio and seawater temperature (Fig. 7c), even though the pattern of temporal variation resembles each other. It is necessary to study the chemical and biological mechanism of the time lag to temperature in future work.

We have conducted the second set of low-resolution analyses on a different *Mytilus* (specimen B, collected from the same place as the first) and temporal variations of Mg/Ca, Sr/Ca, and Ba/Ca ratios are presented in Figures S4a, S4b, and S4c, respectively. There are three peaks of Mg/Ca ratios in the temporal variation. Two of the earlier peaks, located in left hand side (Fig. S4a) are corresponding to the two broad Mg/Ca peaks of specimen A. These peak positions are situated between 13 and 19 mm and between 32 and 40 mm, respectively, very similar to the positions observed in specimen A (Fig. S5). This suggests that the growth rates in both specimens are almost the same. The third peak is observed at between 1 and 3 mm, which is too early to be accounted for by seasonal variation and is not apparent in specimen A (Fig. 4a). Except for these anomalies, tidal control on the Mg/Ca ratio in low-resolution mode is significant. The Sr/Ca ratio of specimen B shows a gradual decrease with the distance from edge (Fig. S4b). However, the rate of change is very small and the tendency is not observed in specimen A (Fig. 4b). The Ba/Ca ratio of specimen B shows a large variation from 0 to 5 mm and then generally remains constant with 2–3 μmol/mol except for spike-type peak at around 10 mm and a broad peak between 14 and 20 mm (Fig. S4c). This variability is consistent with that of specimen A (Fig. 4c) except for the broad peak at 0–5 mm. There is not a one-to-one correlation between the Ba/Ca ratios and heavy precipitation. Recent environmental disturbance and/or physiological reasons after the great tsunami possibly affect the Ba/Ca ratio since the late spring of 2011.

### Tidal cycle and physiological implication

Both analytical modes, high and low resolutions data show a reasonable tidal control on the Mg/Ca ratio of a mussel shell, while there are not valuable variations of Sr/Ca and Ba/Ca ratios related to environmental parameters. The higher resolution of Mg/Ca ratio in Figure 2a shows the diurnal tidal cycle, while lower resolution of that in Figure 4a shows the annual tidal cycles. It is necessary to explain these observations by physiological and/or biochemical effects. Generally, mussels living at the intertidal zone show a tidally controlled growth pattern in their shell, because shell growth is ceased or slow when the bivalves are exposed to air during low tide^11,13,16^. At the time of exposure, mussels keep their valves closed and retract the mantle into the shell. In this condition, there is obviously no supply of Ca from seawater, and calcification rate should be a priori at the minimum. Because of metabolism, the extrapallial fluid (EPF) may become acidic and a part of shell carbonate may be dissolved^16^. Then tiny organic matrices located within the mussel shell may be isolated and/or aggregated, forming a layer of enriched organic matter that selectively binds Mg, but Sr and Ba are not bound^17^. Thus, macro-molecules such as chitin and proteins in EPF may have an important role to increase Mg/Ca ratio in calcite^17^. This calcite layer should correspond to the stained bands by the Mutivei’s solution (Fig. S9) and sulfur enriched lines observed by an EPMA (Figs. 2b and 3b). This layer develops into a growth line that may be also related to the Mg/Ca peaks (Fig. 2c). Such a mechanism could provide a detail interpretation of Mg/Ca changes in bivalve shells, which were interpreted as a response to stress^26^. In this study, the organic layer was only qualitatively qualified by staining and sulfur mapping, but a more detailed discussion will be possible when their quantitative data are available in future analyses.

### Tsunami effects on shell chemistry

At 14:46 JST on March 11, 2011, Tohoku megathrust earthquake (M9.0) occurred off the Pacific coast of Japan with the displaced fault plane, approximately 200 km × 400 km^27^. The M9.0 earthquake induced a great tsunami whose wave height larger than 10 m was estimated in Kamaishi and Miyako City (Fig. S1) and GPS buoy observation in the region (Off Kamaishi and Off Miyako) suggested that there were seven consecutive large waves within six hours^28^. High resolution profiles of Mg/Ca ratios at the tsunami part may record these sea level changes, if the ratio is a proxy of sea level as stated above section in normal tide. There is a large peak of Mg/Ca ratio at the position of 10.311 mm–10.328 mm (Fig. 3c). The large peak height of Mg/Ca ratio shows roughly 10 mmol/mol above the background, which is about 10 times larger than the variations of 0.5–1.5 mmol/mol, caused by a normal tidal cycle. This magnification is comparable with that of excess 10 m high at the tsunami over 1.5 m observed in the maximum variation of sea level due to normal tide (Fig. 7d). Thus, the Mg/Ca ratio of mussel shell may be a promising past tsunami recorder.

In order to prove the tsunami hypothesis, measurements of the second *Mytilus* individual are necessary. However, there is not a precise age model of specimen B by a sclreochronological study. Taking into account of low-resolution data of Ba/Ca ratios of specimen B, there are two spike-type peaks between 10 and 12 mm (Fig. S4). The older one, even though smaller, may roughly correspond to a small peak of Mg/Ca ratio at about 11 mm (see green band in Fig. S4). As a guide of these data, we measured minor (Mg and Sr) and trace (Ba) elements from 10.86 mm to 11.38 mm by a high-resolution mode of 2 μm spot with 2 μm interval. Figure S6a shows that the Mg/Ca ratios vary significantly from 2.21 to 9.18 mmol/mol with the average of 3.79 ± 0.95 mmol/mol (1σ standard deviation, n = 240). There are two peaks higher than one sigma threshold (4.74 mmol/mol). Even though the shape of variations is different from those of specimen A, these anomalies may be due to the great tsunami of March 11, 2011. On the other hand, the Sr/Ca ratios are constant and there is no apparent peak at the position of Mg/Ca anomalies (Fig. S6b). In addition, Ba/Ca ratios are also constant at the position, while there is a large peak at 11.003–11.012 mm, about 0.1 mm younger side (Fig. S6c). This is similar to the location relationship of 0.1 mm for Mg/Ca and Ba/Ca anomalies in specimen A (Fig. 3). These delays of Ba/Ca peaks are converted to 36 h by average growth rate. There may be a physiological explanation, but it should be discussed with a more precise age model in future work.

In conclusion, we have conducted minor and trace elemental analysis of Mediterranean mussel shells collected at the Otsuchi bay, on the Pacific coast of northeastern Japan. The high-resolution Mg/Ca ratios of specimen at inner edge indicates cyclic changes, which may reflect the sea level variation due to tidal force. The Mg enrichment may be derived at low tide when the shell was closed with a low growth rate. This relationship is confirmed by that of a second specimen. The low-resolution data of both specimens covering a whole growth length of shell suggest that the variation of Mg/Ca ratio is attributable to annual tidal cycle. The sea level control on the Mg/Ca ratio is also observed at the time period of the great tsunami induced by the 2011 Tohoku earthquake, where the enrichment and duration of Mg/Ca anomaly are consistent with the characteristics of the tsunami. Therefore, the Mg/Ca ratio of Mediterranean mussel shell is a possible proxy of the past tidal cycle. The mechanism of variation of trace elements in bivalve mollusks cannot be explained by a single mechanism, but rather by a combination of multiple variables. This new proxy for the tidal cycle and the past tsunami is a step toward a better understanding of how to reconstruct paleoenvironmental information from oceanic calcium carbonate.

## Methods

We collected living Mediterranean mussel samples (*Mytilus galloprovincialis*) on the morning of September 6th, 2011 from a mussel bed at 150–200 cm water depth attached to a quay wall in Otsuchi Bay located on the coast of the western North Pacific Ocean in Northeast Japan (Fig. S1). The mussel bed was situated in the intertidal zone as estimated by the animal’s common habitat^13,16^ before the crustal movement caused by the 2011 Tohoku earthquake. Actual coseismic subsidence was not well documented in the region, but it may be approximately 100 cm, estimated in the forearc of Northeast Japan by a model calculation^29^.

Correcting for subsidence after the tsunami, the specimens were probably located at 50–100 cm water depth on September 6th, 2011.

The resin-mounted radial sections of the shell were polished with commercial polishing sheets embedded with fine alumina-grain abrasive (3 M). The polished surface was coated with Pt–Pd vapor and observed with an Electron Probe Micro-Analyzer (EPMA, JXA8230, JEOL, Japan) at the Atmosphere and Ocean Research Institute, University of Tokyo. After EPMA examination, the Pt–Pd coat was removed and coated again by gold to avoid charging during the ion probe analysis. Measurement of minor and trace elements was performed using a NanoSIMS (NS50, Cameca, France) installed at the Atmosphere and Ocean Research Institute, University of Tokyo. LA-ICP-MS analysis on another valve of the same specimen was conducted and their Mn/Ca ratios and oxygen isotopic compositions were reported by a previous work^14^.

In the present work, the analyzed part was the outer calcite layer (prismatic structure) of the mussel shell. There are two types of ion probe measurements conducted using NanoSIMS for mussel samples; high-resolution and low-resolution analyses. For the high-resolution mode, a 100 pA ^16^O^−^ primary ion beam was focused on a 2 μm diameter spot with a 3 μm interval along the maximum growth axis. For the low-resolution mode, a 10 nA ^16^O^−^ primary ion beam was used on a 20 μm diameter spot with a 100 μm interval. At both modes, secondary ions were extracted by an accelerating voltage of 8 kV, introduced into a Mattauch-Herzog type mass analyzer, and ^24^Mg, ^44^Ca, ^88^Sr, and ^138^Ba ions were measured simultaneously by a multi-ion counting system. Isobaric interference was not found except for ^40^Ca_2_^26^Mg^16^O_2_ on the ^138^Ba peak in the mass spectrum of carbonate samples by a mass resolving power of 3100 at 10% peak height. The ^138^Ba/^44^Ca ratio was corrected based on the measured ^24^Mg/^44^Ca and ^138^Ba/^44^Ca ratios^30^. Prior to analysis, the specimen surface was cleaned with an ion beam sputtering. Observed data were calibrated against a natural calcite standard with homogenous and known amounts of Mg, Ca, Sr, and Ba, which were verified by a LA-ICP-MS^30^. The precision associated with the Mg/Ca, Sr/Ca and Ba/Ca ratios are 3%, 3%, and 10% at 2σ, respectively, as estimated by the repeated analysis of the standard.

The other side of the radial section of the shell was embedded as a whole in wider epoxy resin and polished using a plastic sheet abrasive as described above. This specimen was used for the age model to identify the timing of the tsunami on the shell^14^, because the species is known to form a growth line with the enriched organic matter either daily or sub-daily^16,17^. The polished section was stained by Mutvei’s solution, and the images were taken by a digital microscope using the acetate peel technique^31^. It was difficult to count precisely the stained bands with blue color, because their boundaries are sometimes overlapping and not clear. Instead, we have counted the cycle of spring-tide and neap-tide on the photograph (Fig. S7, tidal data in Kamaishi Bay), where spring-tide is characterized by a portion of narrowly spaced growth lines. This method is based on the study on internal growth bands of mussel shell^32^ and a calendar date error would be several days at the maximum in a few years. Experimental details of the age model were given in the previous study^14^.

## Supplementary Information

Supplementary Information.
