# Supplementary material for: Influence of normal tide and the Great Tsunami as recorded through hourly-resolution micro-analysis of a mussel shell
Source: Sci Rep. 2021 Oct 6;11:19874. doi: 10.1038/s41598-021-99361-2 (PMC8494904; doi:10.1038/s41598-021-99361-2)
Supplement: Supplementary file 1 — Supplementary Information. [file 41598_2021_99361_MOESM1_ESM.pdf]

Supplementary Information

Influence of normal tide and the Great Tsunami as recorded through hourly-resolution micro-analysis of a mussel shell

Yuji Sano[1,2*], Tomoyo Okumura[1*], Naoko Murakami-Sugihara[2], Kentaro Tanaka[2], Takanori Kagoshima[3], Akizumi Ishida[4], Masako Hori[5], Glen, T. Snyder[2], Naoto Takahata[2] and Kotaro Shirai[2].

[1]Center for Advanced Marine Core Research, Kochi University, Kochi, Japan

[2]Atmosphere and Ocean Research Institute, University of Tokyo, Chiba, Japan.

[3]Graduate School of Science and Engineering, University of Toyama, Toyama, Japan.

[4]Graduate School of Science, Tohoku University, Sendai, Japan.

[5]Natural Sciences, Osaka Kyoiku University, Osaka, Japan.

*Correspondence should be addressed to Y.S. (e-mail: yuji.sano@kochi-u.ac.jp) and T.O. (e-mail: tomoyook@kochi-u.ac.jp).

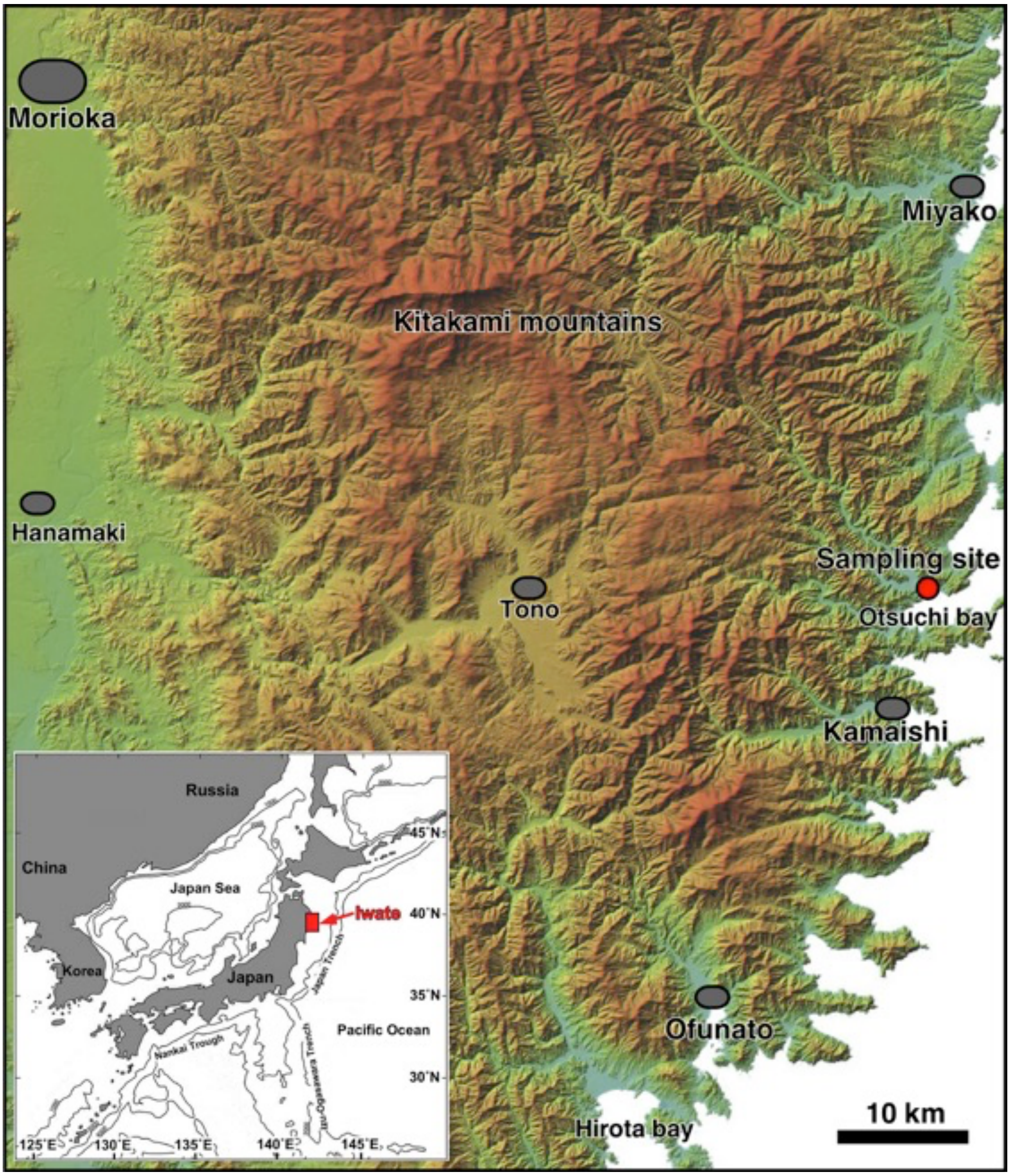

Supplementary Figure 1. Location of mussel shells sampling site in Otsuchi Bay, Iwate prefecture, northeast Japan. Environmental data are taken from Miyako Bay (precipitation), Morioka city (insolation), Hirota Bay (surface seawater temperature), and Kamaishi Bay (sea level change). The inset map shows the location of the Iwate prefecture. The base map is modified from the Digital Japan Portal Web Site, Geospatial Information Authority of Japan (http://maps.gsi.go.jp).

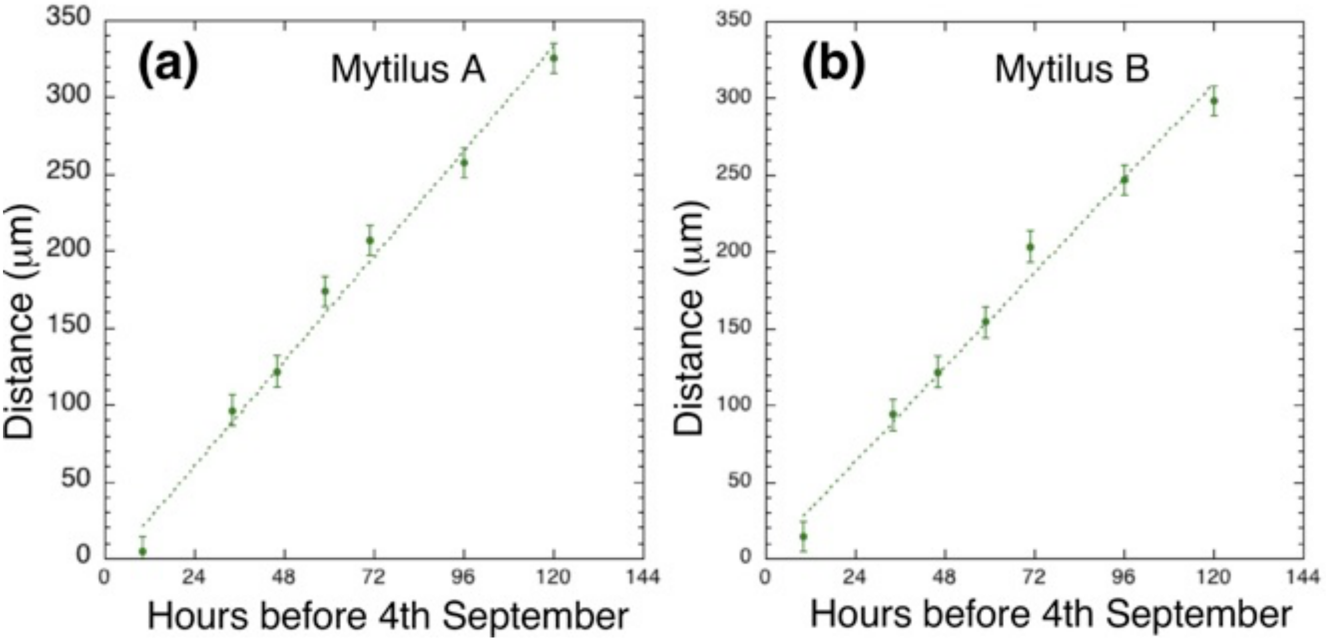

Supplementary Figure 2. Correlation diagram between the time before 4th September 2011 and the distance from the inner edge of a mussel shell. (a) Growth data of *Mytilus* A. Dotted line shows the best fit of y = -7±19 + (2.84±0.28) x where the error is 2σ, R^2=0.988, and MSWD=1.59. (b) Growth data of Mytilus B. Dotted line shows the best fit of y = +2±15 + (2.56±0.22) x where the error is 2σ, R^2=0.987, and MSWD=1.41.

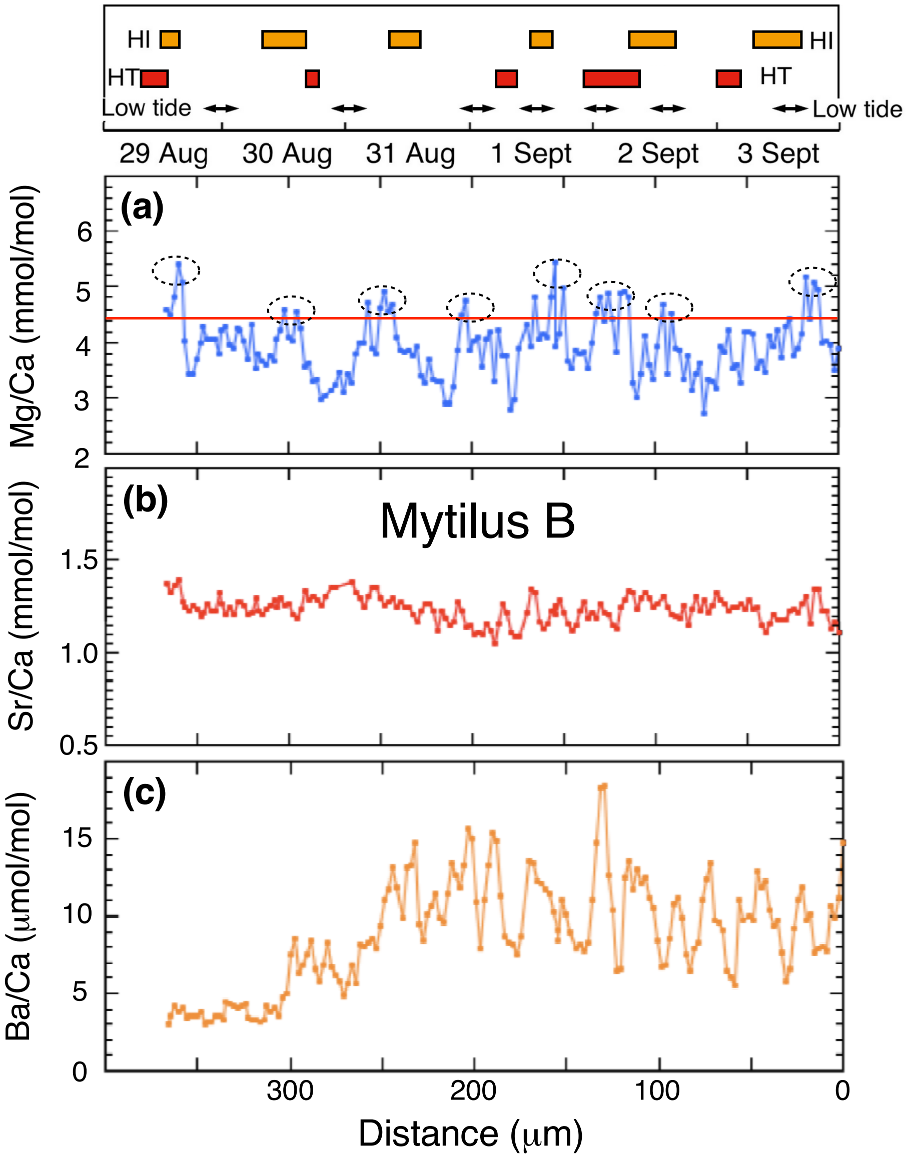

Supplementary Figure 3. High-resolution profile of Mg/Ca, Sr/Ca and Ba/Ca ratios of *Mytilus* B. (a) High-resolution profile of Mg/Ca ratios along the growth line in the inner edge. A red line shows a threshold value. Ovals indicate values higher than the threshold. (b) High-resolution profile of Sr/Ca ratios along the growth line in the inner edge. (c) High-resolution profile of Ba/Ca ratios along the growth line in the inner edge. The simplified scales of the higher insolation (HI), higher temperature (HT), and lower tide period (double-edged arrow) are shown above the profiles. The periods of HI, HT, and lower tide were certified based on the threshold values in Figure 5.

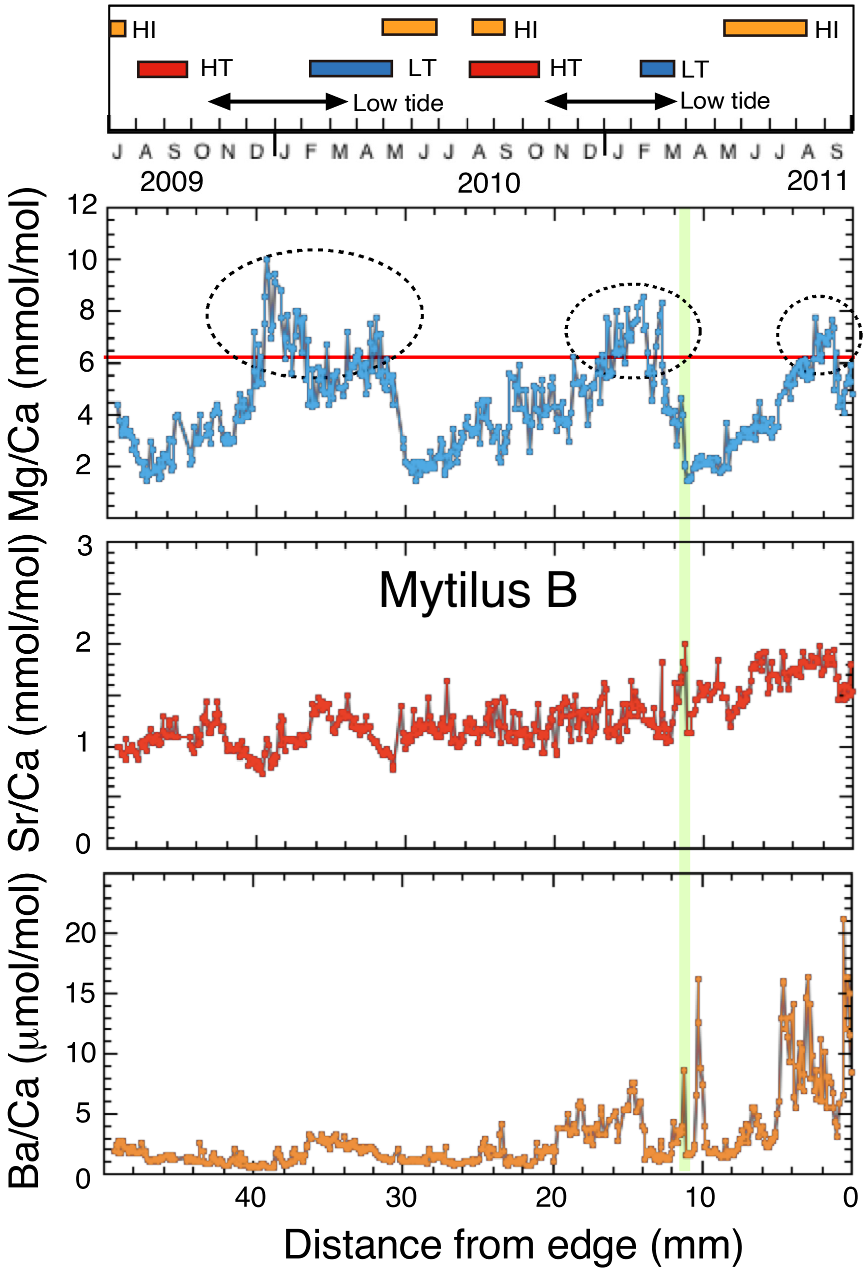

Supplementary Figure 4. Low-resolution profiles of Mg/Ca, Sr/Ca and Ba/Ca ratios of *Mytilus* B. (a) Low-resolution profile of Mg/Ca ratios along the maximum growth line. Horizontal red line and vertical green line show a threshold value and the date of the great tsunami, respectively. Ovals show the data higher than the threshold. (b) Low-resolution profile of Sr/Ca ratios along the growth line. (c) Low-resolution profile of Ba/Ca ratios along the growth line. The simplified scales of the higher insolation (HI), higher temperature (HT), and lower tide period (double-edged arrow) are shown above the profiles. The periods of HI, HT, and lower tide were certified based on the threshold values in Figure 7.

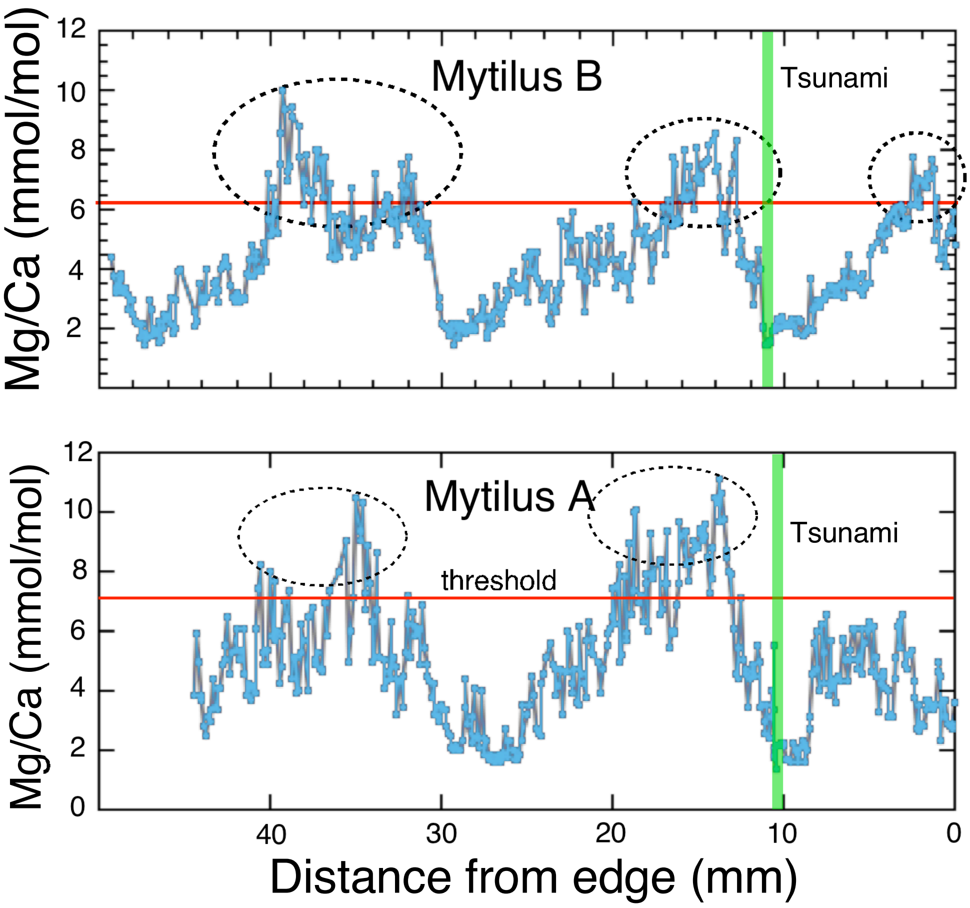

Supplementary Figure 5. Low-resolution profiles of Mg/Ca ratio of the specimen A and B.

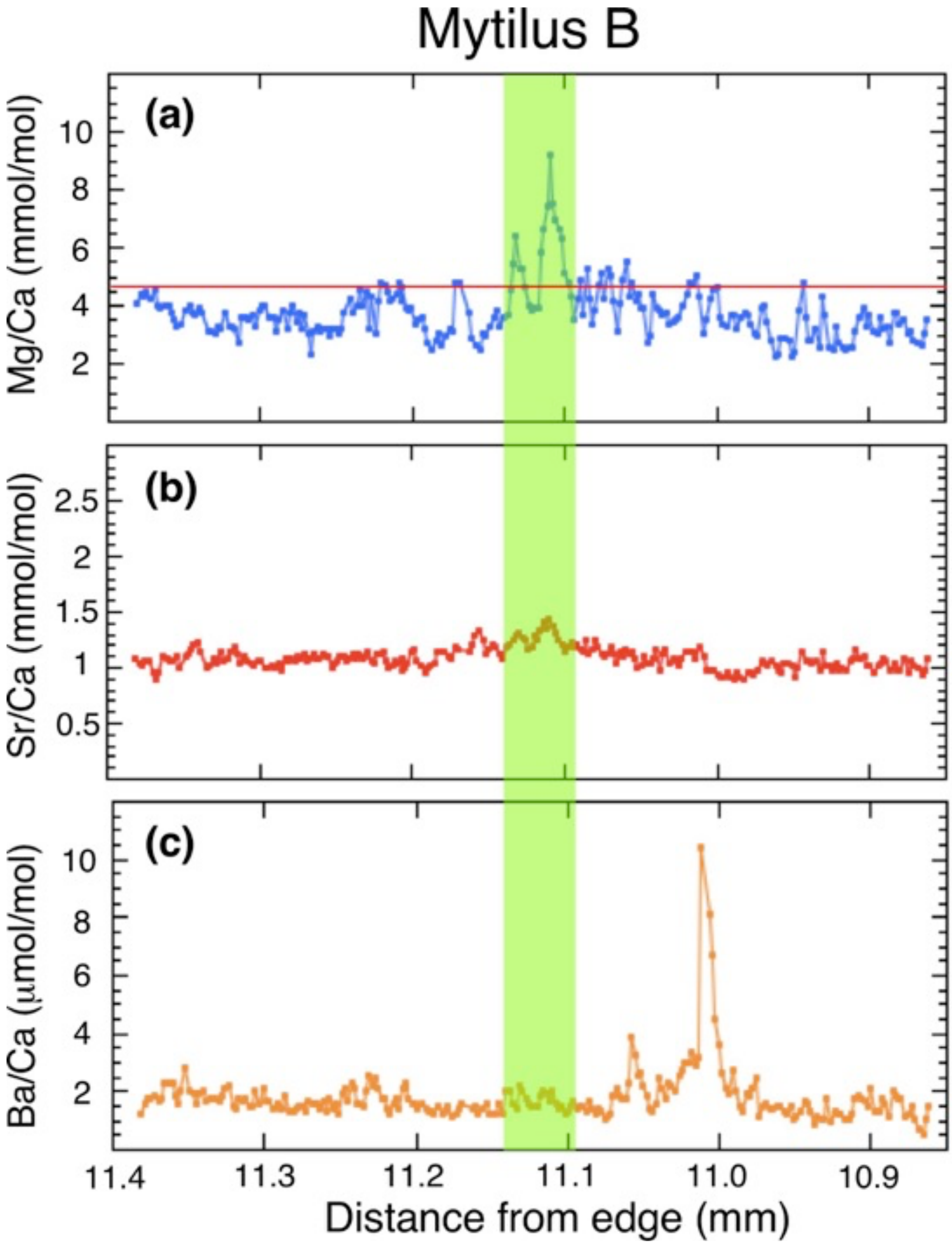

Supplementary Figure 6. High-resolution profiles of Mg/Ca, Sr/Ca and Ba/Ca ratios of *Mytilus* B in tsunami part. (a) High-resolution profile of Mg/Ca ratios across the tsunami part. A red line shows a threshold value. (b) High-resolution profile of Sr/Ca ratios across the tsunami part. (c) High-resolution profile of Ba/Ca ratios across the tsunami part.

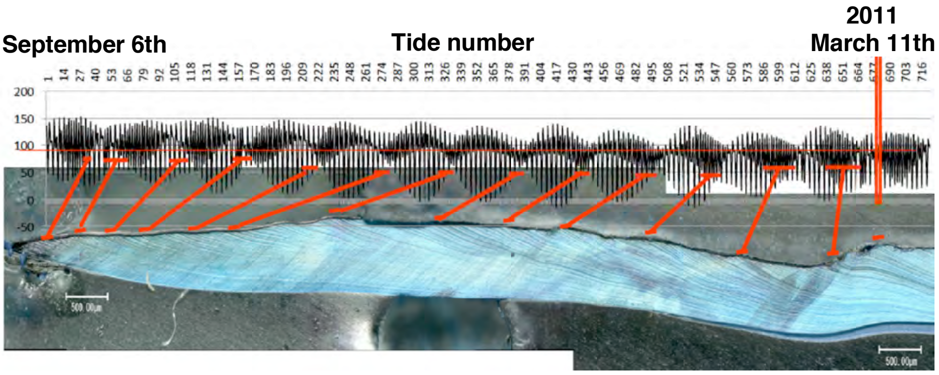

Supplementary Figure 7. Digital microscopy of the section stained by Mutvei's solution to apply a sclerochronology method[14]. Tidal data were calculated by astronomical data in Kamaishi Bay. This figure shows the area corresponding to the square in Figure 1b.
